# Self-Assembled Gefitinib Nanosuspension Prepared via Hummer Acoustic Resonance Technology: Enhanced Dissolution, In Vitro Anticancer Activity and Long-Term Stability

**DOI:** 10.3390/pharmaceutics18030343

**Published:** 2026-03-11

**Authors:** Hai-Li Wu, Ru-Yan Wen, Ling Chen, Zhen-Long Hu, Bao-Yi Qin, Jie-Feng Chen, Meng-Hua Liu, Xuan-Qi Huang, Ning Lin, Qing Chen

**Affiliations:** 1 Institute of Traditional Chinese and Zhuang-Yao Ethnic Medicine, College of Pharmacy, Guangxi University of Chinese Medicine, Nanning 530200, China; wuhaili2023@stu.gxtcmu.edu.cn (H.-L.W.);; 2 Guangxi Innovation Center of Zhuang Yao Medicine, Nanning 530200, China

**Keywords:** gefitinib, hummer acoustic resonance, self-assembly, nanosuspension

## Abstract

**Background**: Gefitinib (Gef) is a first-line epidermal growth factor receptor (EGFR) inhibitor for NSCLC, but its clinical application is limited by poor aqueous solubility and low oral bioavailability. **Methods**: A self-assembled gefitinib nanosuspension (GG-NS) incorporating genistein (Gen) was rapidly developed and optimized via hammer acoustic resonance (HAR) technology. Systematic optimization was conducted using a high-throughput HAR-based process, with particle size, PDI, and zeta potential as key evaluation parameters. Structural and morphological characteristics were analyzed using powder X-ray diffraction (PXRD), thermal analysis, transmission electron microscopy (TEM), and Fourier-transform infrared (FT-IR) spectroscopy. In vitro dissolution behavior and cytotoxicity against A549 lung cancer cells were evaluated. **Results**: Optimal GG-NS with Z-Ave = 223.50 ± 1.53 nm, PDI = 0.239 ± 0.031 and zeta potential = −24.10 ± 0.47 mV was successfully prepared. The nanosuspension remained physically stable for up to five months at both 4 °C and 25 °C. Compared with the raw drugs, GG-NS enhanced the dissolution of gefitinib and genistein in water by 3.76-fold and 13-fold, respectively. In addition, GG-NS showed significantly enhanced cytotoxicity against A549 cells, with a 33.8% higher inhibition rate than the physical mixture after 72 h. **Conclusions**: This study demonstrates, for the first time, that HAR technology enables the rapid fabrication of a self-assembled GG-NS with improved dissolution performance, physicochemical stability, and in vitro anticancer activity, highlighting its promise as an efficient and scalable formulation strategy for poorly soluble anticancer drugs.

## 1. Introduction

Lung cancer remains a formidable and devastating disease, representing the leading cause of cancer-related mortality and the third most common malignancy in terms of incidence [1]. Gefitinib (Gef), an oral administered inhibitor of epidermal growth factor receptor-tyrosine kinase (EGFR-TK), received FDA approval in 2015 as a first-line treatment for non-small cell lung cancer (NSCLC). As a BCS Class II drug, Gef suffers from poor aqueous solubility and low oral bioavailability, consequently leading to compromised clinical efficacy despite its therapeutic potential. Poor aqueous solubility and low bioavailability remain major challenges for the oral delivery of BCS class II/IV drugs in the pharmaceutical industry [2]. Despite its therapeutic potential, Gef is hampered by poor water solubility, low bioavailability and inadequate clinical efficacy. Various formulation approaches, such as nanoparticles [3], solid dispersions [4], lipid-based systems [5], and salts [6], have been investigated to improve its dissolution profile and absorption. Nevertheless, the large-scale production and clinical translation of nanocarrier systems face several persistent challenges, such as complex preparation procedures, poor batch-to-batch reproducibility, and limited physical stability, which greatly limit their clinical application [7]. In contrast, nanosuspensions, which reduce drug particles to the submicron range, offer a simple and effective strategy to enhance dissolution and oral absorption of poorly soluble drugs. Therefore, efficient, cost-effective, and commercially viable nanosuspension technologies are highly needed.

In recent years, extensive research efforts have been directed towards nanosuspension-based delivery systems, which have emerged as a promising and versatile strategy for enhancing the delivery of poorly soluble drugs. Compared to other formulation strategies, nanosuspensions offer distinct advantages, including their capacity to enhance drug solubility, formulation versatility, and compatibility with multiple administration routes, making them one of the most widely utilized strategies for improving the solubility of poorly water-soluble drugs [8]. However, nanosuspensions may face challenges such as stabilizer optimization, physical instability, particle growth during drying, and processing-induced polymorphic transformations [9]. Conventional methods for preparing nanosuspensions are still constrained by several limitations. Current techniques primarily follow top-down and bottom-up pathways [10]. Top-down methods, such as high-pressure homogenization and media milling, rely on mechanical forces (e.g., shear, impact, cavitation) to reduce the particle size of coarse drug crystals. While these processes are robust, scalable, and commonly used in industry, they tend to be energy-intensive, time-consuming, and may involve risks of equipment wear or media contamination. In contrast, bottom-up approaches, like anti-solvent precipitation, generate nanoparticles through controlled nucleation and growth from molecularly dissolved drug solutions. These methods are generally simpler and cost-effective but are heavily dependent on drug solubility in organic solvents. They also face considerable challenges in solvent removal, controlling particle growth, and ensuring long-term stability [11]. Therefore, the development of alternative nanosuspensions preparation technologies that enable rapid processing, precise size control, and scalable manufacturing remains highly desirable.

Hummer Acoustic Resonance (HAR) refers to an innovative, non-contact technology that operates by integrating macroscopic vibrational mixing with microscopic sound field effects [12]. The technology functions on the principle of acoustic resonance energy super-position, where low-frequency sound waves are coupled with mechanical resonance to generate high-frequency vibrations carrying substantial acceleration [13]. This induces a state of chaotic fluidization within the material under acoustic streaming, significantly enhancing the efficiency of particle size reduction. Driven by high-intensity acoustic energy, HAR demonstrates remarkable particle fragmentation efficiency, achieving the target particle size in approximately 2 h. Based on the modular porous platform, HAR enables the parallel, high-throughput preparation of nanosuspensions from trace material quantities, which substantially accelerates formulation screening. This indicates that the critical quality attributes remain consistent when the process is scaled using equipment and containers of varying sizes [14]. This processing time represents a 90% reduction compared to conventional micronization techniques [15]. Unlike conventional wet milling, HAR enables continuous monitoring and dynamic regulation of processing parameters to ensure uniform energy distribution. In addition, the use of isolated containers prevents cross-contamination and reduces cleaning time and downtime [16]. Moreover, its low-shear processing mode minimizes sample damage and particle aggregation, thereby improving nanosuspension stability.

**Figure 1:**
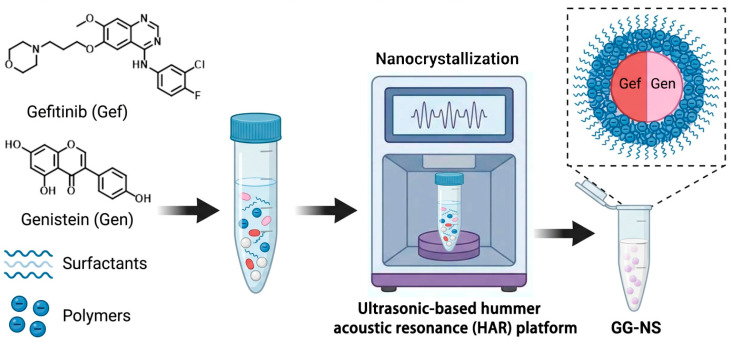
Schematic diagram of the synthesis of GG-NS using the HAR platform.

This study aimed to develop a Gef nanosuspension utilizing an HAR platform. Genistein (Gen), a phytophenol with reported anticancer activity against various cancers and reported synergistic effects with the EGFR tyrosine kinase inhibitor [17,18,19,20], was incorporated as a coformer to construct a Gef nanosuspension (GG-NS). Designed to leverage the pharmacological synergy between Gef and Gen, this system sought to improve the solubility of both drugs, enhance anti-lung-cancer efficacy, and ensure long-term stability. Driven by intermolecular interactions during HAR processing, gefitinib and genistein formed a self-assembled stable nanoscale structure. The research was conducted in a systematic, multi-stage approach. First, a high-throughput screening platform based on HAR technology was employed to evaluate 31 stabilizers or stabilizer combinations, comprehensively exploring the optimal formulation space for GG-NS (Figure 1). Second, the formulation of the GG-NS and the process parameters of HAR technology were optimized using the one-factor-at-a-time (OFAT) method. Third, the optimized GG-NS were systematically characterized in terms of particle size, morphology, crystallinity, thermal behavior, and dissolution performance. Finally, the in vitro anticancer activity of GG-NS was assessed against A549 lung cancer cells. This study provides a novel and potentially scalable strategy for the formulation of poorly water-soluble drugs with improved biopharmaceutical properties.

## 2. Materials and Methods

### 2.1. Materials

Gefitinib (Gef, 98.0% purity) and genistein (Gen, 98.0% purity) were obtained from Meryer Chemical Technology Co., Ltd. (Shanghai, China). Sodium dodecyl sulfate (SDS), polyvinylpyrrolidone K30 (PVP K30), poloxamer 188 (P188), polyvinyl alcohol (PVA), polyethylene glycol 400 (PEG 400), polyethylene glycol 2000 (PEG 2000), hydroxypropyl methylcellulose (HPMC), tween 80, sodium carboxymethyl cellulose (CMC-Na), and methyl cellulose (MC) were provided by Shanghai Macklin Biochemical Co., Ltd. (Shanghai, China). All other reagents and chemicals were of analytical or pharmaceutical grade. The human non-small cell lung cancer cell line A549 was obtained from the Key Laboratory of Guangxi University of Chinese Medicine (Nanning, China). A cell counting kit-8 (CCK-8) was purchased from Shenzhen Mohong Technology Co., Ltd. (Shenzhen, China).

### 2.2. Methods

#### 2.2.1. Preparation of GG-NS

GG-NS were synthesized by the Hummer acoustic resonance equipment (Shenzhen Huasheng Intensification Technology Co., Ltd., Shenzhen, China). 10 mg of API (Gef:Gen = 1:1) was dispersed in a 5 mL centrifuge tube containing stabilizer (5 mg/mL), giving a gefitinib concentration of 3 mg/mL. Zirconia beads (5 mm, 2.3 g) were then added to form the dispersion. First, the optimal resonance frequency was identified in manual mode. Then, the instrument was switched to automatic mode, and resonant acoustic mixing was initiated at a frequency of 61 Hz and a power of 100 W for 1 h. A 20-well platform was employed for stabilizer screening and HAR process optimization of nanosuspensions.

#### 2.2.2. High-Throughput Screening of Stabilizers for GG-NS

A screening of stabilizers was conducted by preparing 31 distinct nanosuspensions using HAR technology. The concentrations in water were 0.5% (*w*/*v*) for the API, 0.5% (*w*/*v*) for single stabilizers, and 0.25% (*w*/*v*) for composite stabilizers. The optimal stabilizer was identified by comparing the hydrodynamic diameter (Z-Ave), polydispersity index (PDI), and zeta potential of nanosuspensions prepared under fixed HAR conditions (80 g acceleration, 1 h).

#### 2.2.3. Optimization of GG-NS Formulation and HAR Process

This study employed the OFAT approach to optimize the GG-NS formulation along with the HAR operating parameters, using the Z-Ave, PDI, and zeta potential as critical quality attributes for evaluation. The OFAT method is operationally simple and practically efficient, making it suitable for the preliminary screening stage of formulations to rapidly identify key influencing factors and their appropriate ranges. In the future, it can be combined with multivariate methods such as design of experiments (DoE) to further investigate factor interactions and response surface patterns, thereby refining and optimizing the formulation. These variables include API concentration, stabilizer ratio, zirconia bead loading, HAR acceleration, and grinding time, as shown in Tables S1–S5 (Supplementary Materials). Following the selection of the optimal formulation and process parameters, six parallel batches of nanosuspensions were prepared to assess both formulation stability and process reproducibility.

### 2.3. Characterization

#### 2.3.1. Particle Characterization

The Z-Ave, PDI, and zeta potential of the GG-NS were determined using a Zetasizer Nano ZS instrument (Malvern, UK). Data from triplicate measurements are presented as mean ± standard deviation.

#### 2.3.2. Morphological Analysis

The surface morphology of GG-NS was examined using a Hitachi HT7700 transmission electron microscope (TEM) (Tokyo, Japan). An appropriate amount of the nanosuspension was diluted with ultrapure water and ultrasonically dispersed for 5 min. Subsequently, 10 μL of the dispersion was dropped onto an ultrathin carbon-coated copper grid that had been glow-discharged in advance. After 2 min of adsorption, any excess liquid was blotted away with filter paper. The air-dried sample was then examined using a TEM (operated at 100 kV) for bright-field imaging.

#### 2.3.3. Powder X-Ray Diffraction (PXRD)

PXRD diffractograms of the sample powder were recorded on a Rigaku MiniFlex 600 diffractometer (Tokyo, Japan) using Cu Kα radiation (λ = 1.540598 Å). An operating voltage of 40 kV and a current of 15 mA were maintained. The samples were scanned on a silicon holder at ambient temperature across a 2θ range of 3° to 50°, employing a scan rate of 10°/s.

#### 2.3.4. Thermal Analyses

Differential scanning calorimetry (DSC) and Thermogravimetry (TG) analysis was conducted over a range of 25–500 °C using the NETZSCH STA 449 instrument (Selb, Germany). Samples were tested (3–5 mg of each) at a heating rate of 10 °C·min^−1^ in a nitrogen atmosphere.

#### 2.3.5. Fourier Transform Infrared Spectroscopy (FT-IR)

FT-IR analysis results were obtained using a Nicolet Nexus 470 FT-IR spectrometer (Waltham, MA, USA). An appropriate amount of the sample was thoroughly ground and homogenized with KBr that had been dried under an infrared lamp. The resulting mixture was then compressed into a transparent pellet using a hydraulic press. Every sample was scanned in a spectral region between 500 and 4000 cm^−1^ with a resolution of 1 scan at 4 cm^−1^.

### 2.4. Dissolution Experiment

Suspensions equivalent to 10 mg of Gef were prepared for Gef, Gen, the physical mixture (Gef+Gen), and GG-NS. Subsequently, 1 mL of each suspension was added to 20 mL of purified water containing 0.1% SDS, sealed, and incubated in a constant-temperature shaking incubator at 37 °C and 100 rpm. At the preset time points (1, 5, 10, 30, 60, 120, 180 min), 1 mL of sample was taken, and an equal volume of preheated 0.1% SDS was immediately supplemented. The samples were filtered through 220 nm microporous membrane, and the drug concentration was detected by high-performance liquid chromatography (HPLC).

### 2.5. HPLC Analysis

The concentrations of Gef and Gen were determined by HPLC, with separations performed on an Agilent 5 TC-C18 column (250 × 4.6 mm; Amstelveen, The Netherlands). The column temperature was maintained at 35 °C. Separation was achieved with a mobile phase comprising methanol and 0.1% triethylamine (80:20, *v*/*v*), delivered at 1.0 mL min^−1^, and the detection wavelengths were set at 250 nm (Gef) and 261 nm (Gen). The concentrations of Gef and Gen were quantified by analyzing the peak areas (using Agilent Chemstation^®^ software B.04.03) from samples that had been filtered through a 220 nm membrane prior to injection.

### 2.6. Cell Viability Assay

The A549 cell lines were utilized to evaluate the antitumor effects of the GG-NS using the CCK-8 assay. Samples were sterilized under ultraviolet irradiation in a biosafety cabinet for 1 h and dispersed in serum-free medium to obtain suspensions at different concentrations. Cells were plated in 96-well plates at a density of 5 × 10^3^ cells/well and allowed to adhere for 12 h. After medium removal, 100 μL of each suspension was added for co-culture. Cell viability was assessed at 48 and 72 h using the CCK-8 assay, with absorbance measured at 450 nm. Untreated cells served as the control. The inhibitory rates were calculated using the following equation:

Inhibitory rate (%) = (1 − ODtreatment/ODcontrol) × 100%.(1)

### 2.7. Stability Assessment of GG-NS

Owing to the inability to immediately dry the prepared nanosuspensions, long-term stability was evaluated. Samples were stored at 4 °C and 25 °C, and changes in particle size and PDI were monitored over 5 months.

### 2.8. Statistical Analysis

Data are reported as the mean ± standard deviation from three independent experimental replicates. The data were statistically analyzed using Analysis of Variance (ANOVA) with GraphPad Prism 9.5.0. Statistical significance was defined as * *p* < 0.05, ** *p* < 0.01, *** *p* < 0.001, and **** *p* < 0.0001.

## 3. Results and Discussion

### 3.1. High-Throughput Screening of Stabilizers for GG-NS

**Figure 2:**
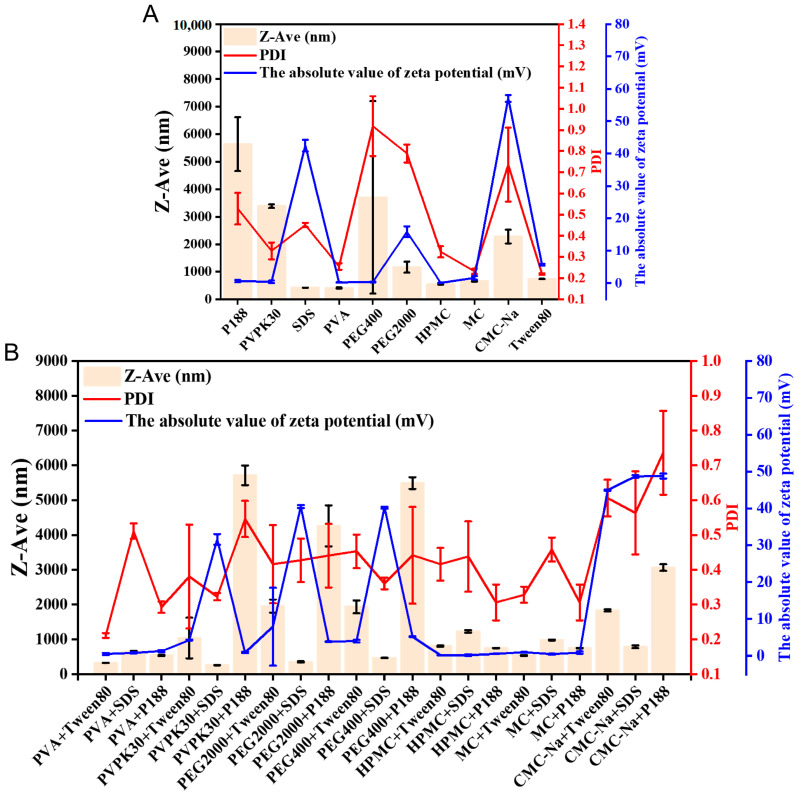
Effect of different stabilizers on the Z-Ave, PDI, and zeta potential of GG-NS: (**A**) single stabilizers; (**B**) combination stabilizers. Data are presented as mean ± SD (*n* = 3).

Nanosuspensions are thermodynamically unstable due to Ostwald ripening and Brownian motion, which increase particle size and broaden distribution, reducing solubility and dissolution rates [21]. The achievement of suitable particle size and storage stability in nanosuspensions largely depends on the appropriate selection of stabilizers and optimization of wet milling conditions. The selection of stabilizers is crucial for the preparation of nanosuspensions, as factors such as drug hydrophobicity, chemical structure, and the molecular weight of the stabilizer may influence their stability [22]. A total of 31 individual stabilizers and stabilizer combinations were screened using HAR technology in a high-throughput manner. Based on electrosteric stabilization principles, three categories of excipients were selected for evaluation: ionic surfactants (SDS), nonionic surfactants (e.g., Tween 80 and P188), and polymers (e.g., CMC-Na, HPMC, MC, PVP K30, PVA, PEG400, and PEG2000) [23]. Delivery Technique Nanosuspension stabilizers act mainly through electrostatic repulsion and steric hindrance. Electrostatic stabilization is provided by charged layers around particles, while steric stabilization results from adsorbed polymers forming protective barriers. Ionic polymers or combinations of polymers and surfactants can integrate both effects, offering efficient and stable protection [24,25]. As shown in Figure 2A, single stabilizers (SDS or PVA) were unable to simultaneously optimize particle size, PDI, and zeta potential because of high polydispersity (PVA) or limited electrostatic stabilization (SDS). By contrast, the PVP K30-SDS combination provided balanced physicochemical properties and was therefore selected as the optimal stabilizing system (Figure 2B). This enhanced stability is attributed to the synergistic steric stabilization provided by PVP K30 and electrostatic repulsion induced by SDS [26].

### 3.2. Optimization and Validation of the GG-NS Formulation and HAR Process Parameters

Wet media milling is a key technique for formulating stable nanosuspensions of diverse poorly water-soluble active compounds. To optimize the nanosuspension process, it is crucial to fine-tune key parameters like milling acceleration, bead loading, and stabilizer concentration, complementing the initial high-throughput stabilizer selection. A high-throughput screening strategy based on HAR technology was thus adopted for the rational formulation optimization of GG-NS. This approach further enhanced formulation optimization efficiency, thereby accelerating the overall drug development process.

#### 3.2.1. Effect of PVPK30 to SDS Ratio

**Figure 3:**
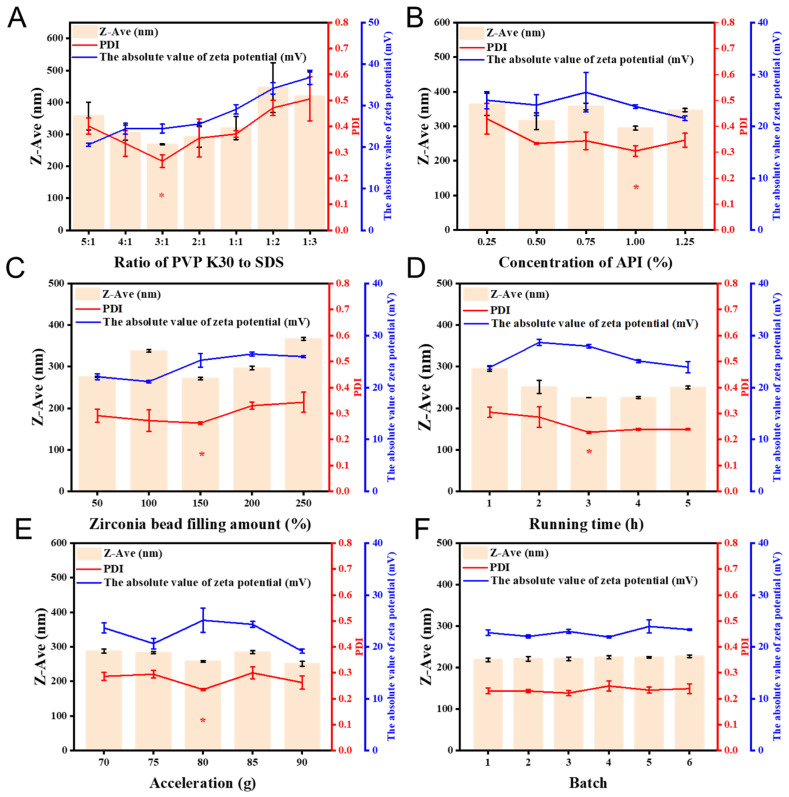
Effect of (**A**) the PVP K30 to SDS ratio; (**B**) concentration of API; (**C**) different zirconia beads filling rate; (**D**) different running time; (**E**) different acceleration; (**F**) different batch on the absolute value of zeta potential, Z-Ave and PDI of GG-NS (* Optimized formulation and processing parameters). Data represent the mean ± SD of three independent experiments.

The effective stabilizer concentration range is defined as being below the CMC, where micelle formation does not occur [27]. It should also be high enough to fully cover the surface of drug particles and maintain dispersion stability. Despite the synergistic stabilizing effect observed between PVP K30 and SDS, an improper ratio of these stabilizers could still induce agglomeration and crystal growth in the nanosuspensions. As shown in Figure 3A, with increasing SDS concentration, the particle size of the nanosuspension gradually decreased, reaching a minimum when PVP K30:SDS = 3:1, accompanied by an increase in zeta potential, which indicates enhanced electrostatic stabilization and improved nanoparticle dispersion. A higher SDS concentration resulted in a larger Z-Ave and reduced stability of the GG-NS, which can be attributed to the increased drug solubility and the consequent acceleration of Ostwald ripening. When PVP K30 was used in excess, the reduced SDS content failed to generate adequate electrostatic repulsion, leading to lower absolute zeta potential values and decreased nanosuspension stability [28]. Based on the evaluation of the three critical quality attributes of GG-NS, a PVP K30 to SDS ratio of 3:1 was identified as the optimal ratio.

#### 3.2.2. Effect of API Concentration

Drug concentration affects bioavailability in a concentration-dependent manner. Moderate levels enhance absorption, while excessive concentrations may cause toxicity and reduce bioavailability. High concentrations can also lower milling efficiency, increase particle size, promote Ostwald ripening, and raise toxicity. Low concentrations help produce smaller and more uniform particles, but may reduce production efficiency and increase cost. Therefore, selecting an appropriate API concentration is essential. As shown in Figure 3B, both Z-Ave and PDI decreased with increasing API concentration, reaching a minimum particle size of 294.8 nm at 1.0% concentration. However, further increases in concentration resulted in a subsequent rise in both parameters. With the drug concentration increases, incomplete adsorption of stabilizers on the particle surfaces may occur, which accelerates crystal nucleus growth and ultimately leads to larger nanocrystal size and particle aggregation [29]. The zeta potential exhibits a dose-dependent decrease, likely due to incomplete stabilizer coverage and the consequent lack of sufficient repulsive forces. However, at 0.75% concentration, the zeta potential is also high. Zeta potential is an important indicator of the electrical double layer. It shows the surface charge of the particles. The sudden increase in zeta potential may be caused by changes in the charge distribution or the adsorption behavior of the drug molecules [30]. These changes can increase the surface charge density, which leads to a higher zeta potential. Consequently, a drug concentration of 1.0% was chosen as the optimal level for subsequent experiments.

#### 3.2.3. Effect of Zirconia Bead Loading

The zirconia bead filling ratio directly affects the efficiency of the wet media milling process. When drug particles come into contact with the grinding media, they experience mechanical stress that initiates and propagates internal cracks, eventually leading to particle fracture [31]. Initially, increasing the zirconia bead filling rate resulted in a gradual reduction in both the average particle size and the PDI of the nanosuspension (Figure 3C). Theoretically, high zirconia bead loading facilitates the production of finer drug nanoparticles. With a fixed milling time, an appropriate zirconia filling ratio is believed to increase the frequency of collisions between the beads and drug particles, thereby enhancing the stress and shear forces applied to the material. Consequently, particle breakage is promoted, leading to a smaller particle size and lower PDI. However, when the filling ratio is too high, the movement of the beads becomes restricted, resulting in reduced milling efficiency. Furthermore, excessive filling tends to generate excess heat during the milling process, increasing the system temperature and intensifying Brownian motion, which ultimately leads to larger particle sizes under the same milling duration [32]. Therefore, a zirconia bead filling rate of 150% was thereby determined to be optimal for subsequent experimentation.

#### 3.2.4. Effect of Grinding Time

Particle breakage is primarily governed by milling time, which promotes mechanical attrition and surface exposure, resulting in reduced particle size and improved dispersion homogeneity [33]. In addition, Prolonged milling promotes more uniform particle dispersion, thereby reducing size variability and enhancing nanosuspension homogeneity. As shown in Figure 3D, 2 h of grinding reduced the average particle size of the drug to below 300 nm, with the PDI decreasing to less than 0.3. Longer milling durations led to a continued decrease in both particle size and PDI. The zeta potential evolved with grinding time, showing an initial rise and a subsequent decline, and peaked in stability at 3 h with a value of −27.9 mV. Milling time also plays a crucial role in determining the zeta potential. Prolonged milling enhanced stabilizers (PVP K30 and SDS) adsorption, increasing the absolute zeta potential to 28.9 mV (>20 mV) and improving physical stability [34]. Consequently, reduced particle size and PDI and increased absolute zeta potential indicated enhanced stability, identifying 3 h as the optimal grinding time.

#### 3.2.5. Effect of HAR Acceleration

In HAR technology, the applied energy is controlled through the adjustment of milling acceleration. With increasing acceleration, particle breakage and collision efficiency are enhanced, resulting in a direct reduction in particle size and a more uniform size distribution. Moreover, intensified collisions lead to the formation of more regular particle morphology. Consequently, the physical stability of the nanosuspension is significantly improved, and particle aggregation and Ostwald ripening are effectively suppressed. As shown in Figure 3E, increasing HAR acceleration led to a progressive reduction in nanosuspension particle size. The minimum particle size and PDI were obtained at an acceleration of 80 g. At this stage, while the absolute value of the zeta potential magnitude (>20 mV) indicated good stability of the nanosuspensions, further increases in acceleration led to a rise in mean particle size, broader particle size distribution, and apparent instrument overload. Excessive milling acceleration was found to exert dual adverse effects due to thermal influences. On the one hand, the increase in system temperature enhanced the solubility of API, thereby accelerating particle growth through Ostwald ripening and reducing the uniformity of the particle size distribution [35]. On the other hand, the balance between fragmentation and aggregation was disturbed, accompanied by intensified crystal damage and potential product contamination. Consequently, the optimal HAR acceleration was established at 80 g for subsequent investigations.

### 3.3. Physicochemical Characterization

#### 3.3.1. Morphology Analysis

**Figure 4:**
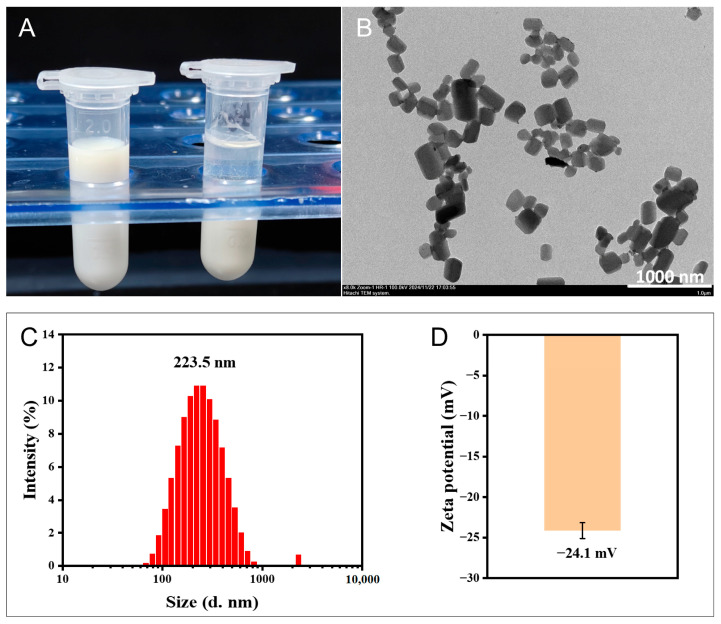
Characterization of GG-NS: (**A**) Appearance of freshly prepared GG-NS (left) and GG-water (right); (**B**) TEM image (8000×); (**C**) particle size distribution; (**D**) zeta potential. Zeta potential measurements were performed in triplicate and are reported as mean ± SD; (d. nm) = particle diameter in nanometers.

The particle size, morphology, dispersibility, and surface charge of the optimized GG-NS were characterized using TEM and a Malvern particle size analyzer. As shown in Figure 3F, the six batches of GG-NS prepared in parallel exhibited consistent quality, indicating that the optimized formulation process enables the stable and reproducible production of GG-NS. As shown in Figure 4A, the optimal GG-NS exhibits uniform white color, while the suspension without stabilizer settles within ten minutes. This may be due to the fact that the added stabilizer is adsorbed on the surface of the nanoparticles, reducing the collision and aggregation between the particles through electrostatic repulsion, thereby giving the nanoparticles better stability. As shown in Figure 4B, GG-NS was a bulk-like crystal with uniform particle size distribution. Acoustic energy can be uniformly distributed throughout the mixing system, resulting in a narrower particle size distribution [36]. Meanwhile, rapid stabilizer adsorption onto nascent surfaces, driven by intensive mixing, enhances surface charge and zeta potential stability. The particle size of GG-NS predominantly falls within the range of 200 nm to 500 nm, aligning with the measurements obtained from the laser particle size analyzer. The nanosuspensions nanoparticles exhibited a mean particle size of 223.50 ± 1.53 nm with narrow size distribution (PDI = 0.239 ± 0.031) and a zeta potential of −24.10 ± 0.47 mV, indicating good stability (Figure 4C,D). SDS and PVP K30 function jointly to establish a composite stabilization barrier on GG-NS, preventing crystal growth and particle aggregation through electrostatic and steric mechanisms.

#### 3.3.2. PXRD Analysis

**Figure 5:**
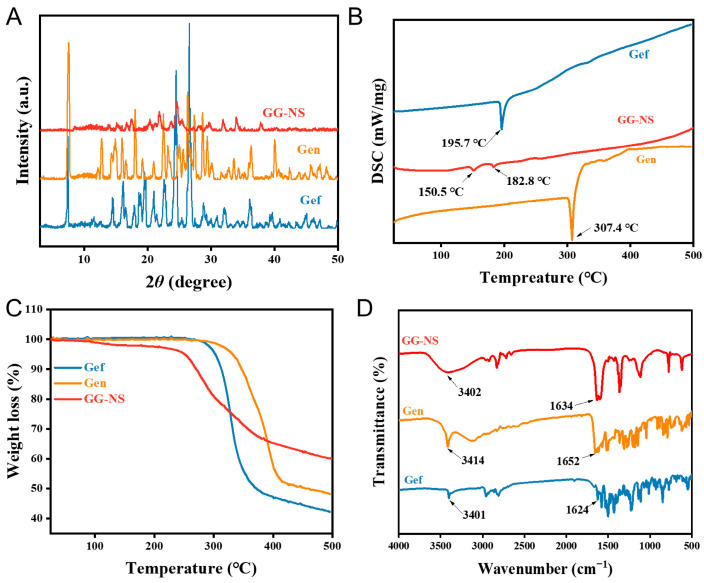
(**A**) PXRD diffractograms; (**B**) TG thermograms; (**C**) DSC thermograms and (**D**) FT-IR spectra of Gef, Gen, and GG-NS.

The powder X-ray diffraction (PXRD) diffractograms of Gef, Gen, and GG-NS are displayed in Figure 5A. It can be seen that GG-NS has several distinct diffraction peaks at 17.48°, 20.35°, 21.88°, 21.86°, 31.86°, 34.01°, each of which is significantly different from the PXRD diffractograms of their parental compounds. These findings indicate that the intermolecular interactions and molecular packing within the GG-NS nanoparticles were altered, arising from the self-assembly of Gef and Gen mediated by intermolecular weak interactions. Such self-assembly is expected to modify the physicochemical properties of the system, including solubility and bioavailability, thereby potentially influencing its pharmacological performance. Compared with the characteristic diffraction peaks of raw Gef and Gen, the PXRD patterns of GG-NS showed slightly reduced intensities, suggesting decreased crystallinity after wet milling.

#### 3.3.3. Thermal Analysis

To explore the thermodynamic behavior of the GG-NS, thermogravimetric analysis (TG) and differential scanning calorimetry (DSC) were conducted. As shown in Figure 5B, pure Gef and Gen exhibited distinct melting endotherms at 195.7 °C and 307.4 °C, respectively. In contrast, GG-NS displayed a broad endothermic event at 150.5 °C followed by a sharp melting peak at 182.8 °C, which differed markedly from those of the individual components, indicating the formation of a new solid phase. Notably, this change in melting point indicates an alteration in intermolecular interactions and molecular packing within the nanoparticles. A small solvent peak at 150.5 °C on the DSC curve is evident, in conjunction with a concurrent mass loss step on the TG profile, indicating that water molecules were involved in the formation of self-assembly (Figure 5C). Additionally, compared to the raw materials, the wet-milled drug particles exhibited weaker endothermic melting peaks, which can be attributed to reduced crystal size, increased surface energy, and the introduction of lattice imperfections during the milling process, in agreement with the PXRD observations.

#### 3.3.4. FT-IR Analysis

Fourier transform infrared spectroscopy (FT-IR) can indirectly reveal the formation of self-assembled structures through changes in molecular vibrational modes. The FT-IR spectra of Gef, Gen, and the GG-NS are depicted in Figure 5D. The distinctive vibrational band of Gef were precisely identified, including the vibrational band at 3401 cm^−1^ associated with the N-H stretching vibration, and the vibrational bands observed at 1624 cm^−1^ corresponding to the C=N vibrations of the pyrimidine ring [37]. The strong absorption bands of Gen at 3414 and 1652 cm^−1^ are attributed to the O-H and C=O stretching vibration. FTIR analysis of GG-NS revealed a shift of the combined O-H/N-H vibrational band to 3402 cm^−1^, accompanied by a red shift of the Gen C=O stretching vibration from 1652 cm^−1^ to 1631 cm^−1^, collectively suggesting the formation of hydrogen bonds between Gef and Gen upon self-assembly. In addition, the stretching vibration of the O-H and N-H bonds becomes broader, indicating the enhancement of chemical bonds [38]. The FT-IR results confirmed the successful self-assembly of Gef and Gen within the GG-NS, as evidenced by characteristic vibrational band shifts and broadening. In particular, the red shift of the absorption band suggests that hydrogen bonding between O-H/N-H and C=O groups plays a key role in the self-assembly of the two drug molecules [39].

### 3.4. Dissolution Studies

**Figure 6:**
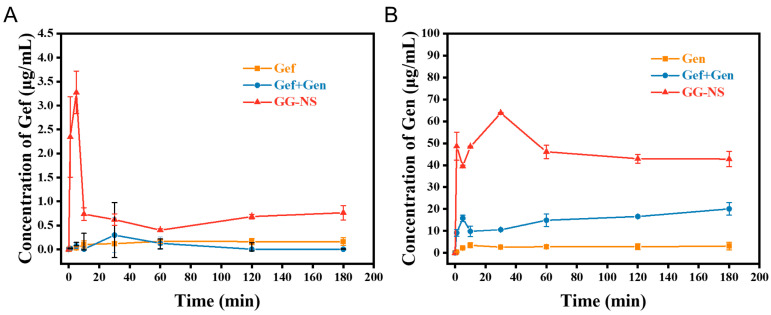
In vitro dissolution profiles of Gef, Gen, and GG-NS: (**A**) Gef concentration; (**B**) Gen concentration. All measurements were conducted in triplicate and are presented as mean ± SD.

A key advantage of drug nanosuspension formulations is their ability to markedly enhance the dissolution rate. The dissolution behaviors of Gef, Gen, Gef+Gen, and GG-NS were investigated in 0.1% SDS. As shown in Figure 6A, the nanosuspension formulation significantly enhanced solubility, exhibiting a 3.8-fold increase compared with Gef, whereas the physical mixture displayed a solubility profile similar to that of Gef. The improvement can be ascribed to the markedly increased surface area, reduced diffusion distance, and higher saturation solubility associated with nanosizing, which collectively promoted faster dissolution. The further enhancement in gefitinib dissolution from the nanosuspension formulation is consistent with the “spring–parachute” phenomenon, where rapid generation of a supersaturated state (spring effect) during dissolution is kinetically maintained (parachute effect), resulting in prolonged elevated drug concentration and enhanced apparent solubility [40]. This model has been used to describe dissolution behavior in poorly water-soluble drug systems and underscores the importance of supersaturation maintenance for improved drug release performance. Interestingly, compared with pure genistein, and nanosizing further enhanced dissolution up to 13-fold, as shown in Figure 6B. Although purified water containing 0.1% SDS enables standardized comparison of dissolution behavior, it does not fully mimic gastrointestinal conditions. Therefore, biorelevant media (simulated gastric fluid and simulated intestinal fluid) will be used in future studies to further evaluate the in vivo-relevant dissolution performance of GG-NS.

### 3.5. In Vitro Cytotoxicity

**Figure 7:**
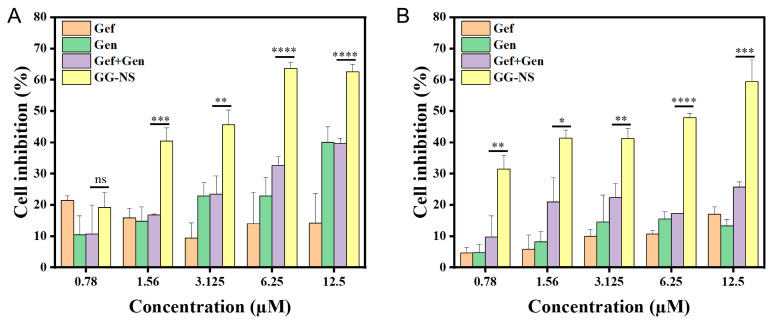
Inhibition rates of A549 cells treated with Gef, Gen, Gef+Gen, and GG-NS at different incubation times: (**A**) 48 h; (**B**) 72 h. Data are shown as mean ± SD (*n* = 3), with statistical significance indicated as ns (non-significant), * *p* < 0.05, ** *p* < 0.01, *** *p* < 0.001, and **** *p* < 0.0001 relative to the Gef+Gen group.

To investigate the effect of optimized GG-NS on the antitumor activity of Gef, the cytotoxicity of Gef, Gen, GG-NS, and their physical mixture against A549 cells was evaluated using the CCK-8 assay. The compounds were tested on human non-small cell lung cancer A549 cells at concentrations ranging from 0.78 to 12.5 μM (Figure 7). Compared with free drugs and their physical mixture, GG-NS exhibited significantly enhanced inhibitory activity. In particular, GG-NS showed markedly higher cytotoxicity than Gef+Gen at concentrations of 6.25–12.5 μM, indicating that the nanosuspension formulation improved drug performance, likely by enhancing dissolution. Notably, GG-NS achieved the highest inhibition rates at nearly all tested concentrations and time points, reaching approximately 65% at 12.5 μM after 72 h, which was nearly 33.8% higher than that of free Gef (Figure 7B). This superior performance may be attributed to the reduced particle size and increased surface area of GG-NS, which facilitate rapid dissolution and promote drug availability to tumor cells [41]. Collectively, these results demonstrate that GG-NS effectively overcomes the solubility limitation of gefitinib and enhances its cytotoxic efficacy, providing a promising strategy for improving the therapeutic performance of poorly soluble anticancer drugs.

### 3.6. Stability Assessment

**Figure 8:**
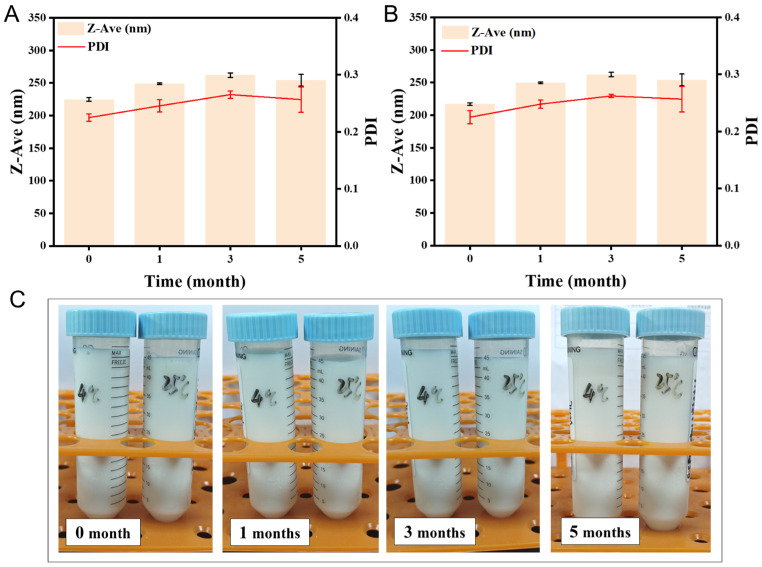
Long-term stability evaluation of GG-NS: (**A**) 4 °C and (**B**) 25 °C, with representative appearance shown in (**C**). Data are expressed as mean ± SD (*n* = 3).

The physical stability of GG-NS was evaluated by storing samples at 4 °C and 25 °C for 5 months, with periodic monitoring of particle size and PDI. As presented in Figure 8, only minor changes in particle size (<10 nm) were observed at both temperatures, with PDI values maintained below 0.3. The five-month storage stability of GG-NS suspension, with minimal particle aggregation, underscores the suitability of the PVP K30-SDS combination as the optimal stabilizer. The PDI progressively increased during storage at 25 °C compared to 4 °C, presumably because of intensified nanoparticle movement and incipient aggregation at the higher temperature. Sayyad et al. (2025) [42] developed a gefitinib nanosuspension using high-pressure homogenization, achieving an average particle size of 157 ± 18.77 nm. Although this particle size was smaller than that obtained in the present study, their formulation exhibited limited stability, with the lyophilized powder remaining stable for only three months. In contrast, the GG-NS prepared using hummer acoustic resonance technology demonstrated superior stability for up to five months, highlighting the advantages of the present formulation strategy. Nevertheless, further studies are still required to evaluate the long-term stability of GG-NS under diverse storage and environmental conditions, which will be essential for its future development and clinical translation.

## 4. Conclusions

In this study, a self-assembled GG-NS was synthesized for the first time using hummer acoustic resonance (HAR) technology. A high-throughput strategy was employed to rapidly and systematically optimize both formulation variables and HAR processing parameters, resulting in a stable nanosuspension with Z-Ave = 223.50 ± 1.53 nm, PDI = 0.239 ± 0.031 and zeta potential = −24.10 ± 0.47 mV. Using HAR technology, the optimized formulation showed high reproducibility, with stable particle size and PDI for up to five months at 4 °C and 25 °C. Compared with gefitinib raw material and physical mixtures, the nanosuspension significantly improved drug solubility and enhanced antitumor activity against A549 lung cancer cells. The superior dissolution and biological performance of GG-NS demonstrates the advantages of nanosuspensions prepared by HAR technology. Nanotechnology can enhance drug solubility and promote absorption by reducing particle size and increasing specific surface area, while their stability may facilitate reliable drug release in vivo and sustained therapeutic efficacy. Accordingly, the improved physicochemical properties of GG-NS are expected to translate into favorable in vivo performance. Future studies will focus on pharmacokinetic and antitumor evaluations to validate its clinical potential.

## Data Availability

All data needed to support the conclusions in the paper are presented in the manuscript. Additional data related to this paper may be requested from the corresponding author upon request.

## Supplementary Materials

The following supporting information can be downloaded at: https://www.mdpi.com/article/10.3390/pharmaceutics18030343/s1, Table S1: Specific formulations and process parameters for the PVP K30 to SDS ratio screening experiment; Table S2: Specific formula and process parameters for the API concentration screening experiment; Table S3: Specific formulations and process parameters for the zirconia beads filling rate screening experiment; Table S4: Specific formulations and process parameters for the running time screening experiment; Table S5: Specific formulations and process parameters for the acceleration screening experiment.

## Abbreviations

The following abbreviations are used in this manuscript:

Gef	Gefitinib
HAR	Hummer acoustic resonance
GG-NS	Gefitinib nanosuspension
TG	Thermogravimetric
DSC	Differential Scanning Calorimetry
PXRD	Powder X-ray diffraction
FT-IR	Fourier-transform infrared spectroscopy
NSCLC	Non-small cell lung cancer
EGFR	Epidermal growth factor receptor
API	Active pharmaceutical ingredients
TEM	Transmission electron microscopy
PDI	Polydispersity index
