# Supplementary material for: Self-Assembled Gefitinib Nanosuspension Prepared via Hummer Acoustic Resonance Technology: Enhanced Dissolution, In Vitro Anticancer Activity and Long-Term Stability
Source: Pharmaceutics. 2026 Mar 11;18(3):343. doi: 10.3390/pharmaceutics18030343 (PMC13028922; doi:10.3390/pharmaceutics18030343)
Supplement: Supplementary file 1 [file pharmaceutics-18-00343-s001.zip › pharmaceutics-4131787-supplementary.pdf]

pharmaceutics

Supplementary Materials: Self-Assembled Gefitinib Nanosuspension Prepared via Hummer Acoustic Resonance Technology: Enhanced Dissolution, In Vitro Anticancer Activity and Long-Term Stability

Hai-Li Wu, Ru-Yan Wen, Ling Chen, Zhen-Long Hu, Bao-Yi Qin, Jie-Feng Chen, Meng-Hua Liu, Xuan-Qi Huang, Ning Lin and Qing Chen

Table S1. Specific formulations and process parameters for the PVP K30 to SDS ratio screening experiment.

API concentration (w/v)	PVP K30 to SDS(v/v)	Filling rate (%)	Acceleration (g)	Running time (h)
0.5%	5:1	100%	80	1
0.5%	4:1	100%	80	1
0.5%	3:1	100%	80	1
0.5%	2:1	100%	80	1
0.5%	1:1	100%	80	1
0.5%	1:2	100%	80	1
0.5%	1:3	100%	80	1

Table S2. Specific formula and process parameters for the API concentration screening experiment.

API concentration (w/v)	Stabilizer concentration (w/v)	Filling rate (%)	Acceleration (g)	Running time (h)
0.25%	0.5%	100%	80	1
0.5%	0.5%	100%	80	1
0.75%	0.5%	100%	80	1
1%	0.5%	100%	80	1
1.25%	0.5%	100%	80	1

Table S3. Specific formulations and process parameters for the zirconia beads filling rate screening experiment.

API concentration (w/v)	Stabilizer concentration (w/v)	Filling rate (%)	Acceleration (g)	Running time (h)
1%	0.5%	50%	80	1
1%	0.5%	100%	80	1
1%	0.5%	150%	80	1
1%	0.5%	200%	80	1
1%	0.5%	250%	80	1

Table S4. Specific formulations and process parameters for the running time screening experiment.

API concentration (w/v)	Stabilizer concentration (w/v)	Filling rate (%)	Acceleration (g)	Running time (h)
1%	0.5%	150%	80	1

1%	0.5%	150%	80	2
1%	0.5%	150%	80	3
1%	0.5%	150%	80	4
1%	0.5%	150%	80	5

Table S5. Specific formulations and process parameters for the acceleration screening experiment.

API concentration (w/v)	Stabilizer concentration (w/v)	Filling rate (%)	Acceleration (g)	Running time (h)
1%	0.5%	150%	70	3
1%	0.5%	150%	75	3
1%	0.5%	150%	80	3
1%	0.5%	150%	85	3
1%	0.5%	150%	90	3
